# Colorectal Cancer Screening in Switzerland: Cross-Sectional Trends (2007-2012) in Socioeconomic Disparities

**DOI:** 10.1371/journal.pone.0131205

**Published:** 2015-07-06

**Authors:** Stacey A. Fedewa, Stéphane Cullati, Christine Bouchardy, Ida Welle, Claudine Burton-Jeangros, Orly Manor, Delphine S. Courvoisier, Idris Guessous

**Affiliations:** 1 Emory University, Department of Epidemiology, Atlanta, GA, United States of America; 2 American Cancer Society, Atlanta, GA, United States of America; 3 Unit of population epidemiology, Department of Community Medicine, Primary Care and Emergency Medicine, University Hospitals of Geneva, Geneva, Switzerland; 4 Geneva Cancer Registry, Global Health Institute, University of Geneva, Geneva, Switzerland; 5 Institute of Social and Preventive Medicine, Lausanne University Hospital, Lausanne, Switzerland; 6 Department of Sociology, University of Geneva, Geneva, Switzerland; 7 School of Public Health and Community Medicine, Hebrew University-Hadassah, Jerusalem, Israel; 8 Division of Clinical Epidemiology, University Hospitals of Geneva, Geneva, Switzerland; 9 Division of Chronic Disease, University Institute of Social and Preventive Medicine, Lausanne University Hospital, Lausanne, Switzerland; The University of Texas MD Anderson Cancer Center, UNITED STATES

## Abstract

**Background:**

Despite universal health care coverage, disparities in colorectal cancer (CRC) screening by income in Switzerland have been reported. However, it is not known if these disparities have changed over time. This study examines the association between socioeconomic position and CRC screening in Switzerland between 2007 and 2012.

**Methods:**

Data from the 2007 (n = 5,946) and 2012 (n = 7,224) population-based Swiss Health Interview Survey data (SHIS) were used to evaluate the association between monthly household income, education, and employment with CRC screening, defined as endoscopy in the past 10 years or fecal occult blood test (FOBT) in the past 2 years. Multivariable Poisson regression was used to estimate prevalence ratios (PR) and 95% Confidence Intervals (CI) adjusting for demographics, health status, and health utilization.

**Results:**

CRC screening increased from 18.9% in 2007 to 22.2% in 2012 (p_adjusted_: = 0.036). During the corresponding time period, endoscopy increased (8.2% *vs*. 15.0%, p_adjusted_:<0.001) and FOBT decreased (13.0% *vs*. 9.8%, p_adjusted_:0.002). CRC screening prevalence was greater in the highest income (>$6,000) vs. lowest income (≤$2,000) group in 2007 (24.5% *vs*. 10.5%, PR:1.37, 95%CI: 0.96-1.96) and in 2012 (28.6% *vs*. 16.0%, PR:1.45, 95%CI: 1.09-1.92); this disparity did not significantly change over time.

**Conclusions:**

While CRC screening prevalence in Switzerland increased from 2007 to 2012, CRC screening coverage remains low and disparities in CRC screening by income persisted over time. These findings highlight the need for increased access to CRC screening as well as enhanced awareness of the benefits of CRC screening in the Swiss population, particularly among low-income residents.

## Introduction

In 2012, 1,361,000 people were diagnosed with colorectal cancer (CRC) and 694,000 people died from CRC worldwide. [1] The majority of CRCs are diagnosed in developed countries. For example, world-age-standardized CRC incidence in New Zealand, Australia, Canada, United States and Western Europe, including Switzerland, exceeds 25 cases per 100,000 people. [1] In order to reduce CRC incidence and mortality, fecal occult blood testing (FOBT) annually, sigmoidoscopy every 5 years with hemoccult periodically or colonoscopy every 10 years are recommended for average-risk adults between 50–75 years by European Panel [2] and the US Preventive Services Task Force. [3] There is no national organized CRC screening program in Switzerland and CRC screening among Swiss residents is low. According to the 2007 Swiss Health Interview Survey (SHIS), only 13% of adults ≥ 50 years had a hemoccult test or endoscopy in the past 5 years for screening reason. [4] In addition to suboptimal CRC screening utilization in Switzerland, social inequalities have been noted where adults highest income bracket were 70% more likely to receive a screening endoscopy.[4] However, temporal patterns of CRC screening patterns by socioeconomic status have not been investigated, which is of interest given a recent Swiss study reporting growing disparities in healthcare renunciation between 2007 and 2010 [5] and projected increases in CRC incidence.[6] The last population based SHIS in 2012 provides an opportunity to examine temporal patterns of CRC screening by socioeconomic status, measured by household income, education, and employment status. This study examines the association between socioeconomic position and CRC screening prevalence as well as potential changes of social disparities between 2007 and 2012 in the SHIS.

## Methods

### Survey design

The SHIS is a cross-sectional survey repeated every 5 years since 1992 and conducted by the Swiss Federal Statistical Office.[7] It is designed to be representative of all residents’ aged 15 years and older living in Switzerland, which are randomly selected following a two-stage stratified sampling strategy. First, respondents were contacted by phone and interviewed using computer–assisted telephone interview. Second, respondents received at home a self-administered questionnaire (either paper or online). The response rate was 66.2% in 2007 and 53.1% in 2012.[8] Data are de-identified and according to Swiss law, de-identified routine health data do not require approval by ethics committees.[9] The present study included the 2007 and 2012 waves because CRC screening questions were introduced only in 2007 and 2012 is the most recent survey year available. We only examined respondents aged 50 to 75 years old (N = 16,059), according to U.S and European CRC screening recommendations. [2, 3] We excluded respondents with missing data on CRC screening use (N = 524), socio-economic or–demographic profile (N = 1,290), health status (N = 175), and health service use (N = 900). The final study population included 13,170 individuals.

### Dependent variables

Respondents were asked several CRC screening related questions as follows: 1) “Have you ever had a hemoccult test?” 2) “Have you ever had a visual examination of the colon? (Endoscopy, sigmoidoscopy, colonoscopy)?” If respondents answered yes to either question, the date and the reason (diagnostic, screening or screening program) for the last test were ascertained. Hemoccult tests included all FOBTs (guaiac, hemoccult) as well as fecal immunochemical test (FIT). The analysis was restricted to screening related test. We considered only tests performed within 2 years for the hemoccult and 10 years for endoscopy because sigmoidoscopy is rarely performed (<4% of all CRC screenings) in Switzerland. [10] Three separate outcomes were considered: hemoccult in the past 2 years, endoscopy in the past 10 years, and any CRC screening (hemoccult in the past 2 years or endoscopy in the past 10 years).

### Independent variables

Three indicators of socioeconomic position were used: income (≤$2,000, $2,001–4000, $4,001-$6,000, >$6,000), education (compulsory, secondary, and tertiary), and employment (employed *vs*. out of the labor force). Among employed respondents, occupational class (liberal, intermediate, non-manual professions, independent/artisans, overseer/qualified worker, skilled worker) were also considered. Household income was weighted by the number of persons living in the household. In October 2014, 1 US dollar (USD) corresponded to 1 Swiss Franc (CHF). Educational levels generally corresponded to the International Standard Classification of Education 1997[11]: compulsory education corresponded to primary and lower secondary education (approximately 9 years of education starting at age 4 or 5), secondary education includes additional specialized training including vocational training (approximately 1–3 years of additional education), and tertiary included more theory-based and specialized degrees which correspond to bachelors, masters and doctoral degrees (approximately an extra 1–8 years of education). Occupational class was based on the Erikson, Goldthorpe and Portocarero social class scheme [12] which classified occupation based on job duties, setting/environment and management responsibilities.

Other sociodemographic characteristics included sex, age (50–64, 65–75), marital status (single, married/registered partnership, widow, divorced/separated/registered partnership dissolved), and citizenship (Swiss versus not Swiss). Geographic residence was grouped as: metropolitan areas, medium size urban areas, small size urban areas, and rural areas.

Health status covariates included self-rated health (SRH), physical symptoms, psychological distress, health service use, smoking (current versus former/never smoker) and body mass index (BMI). The SRH questions varied between the two surveys. In 2007, respondents were asked “How is your health in general?” and response categories included very good, good, good enough, bad, and very bad. In 2012, respondents were asked “How is your health condition in general?” and response categories included very good, good, average, bad and very bad. SRH was categorized as very good, good, average/good enough, bad and very bad to capture the different response categories over the two surveys. Respondents were asked about the presence and frequency of the following eight physical symptoms in the past four weeks: backache, general weakness, stomach ache/bloating, diarrhea/constipation, insomnia, headache, cardiac arrhythmia, chest pain. The total number of physical symptoms in the past four weeks was classified as: no or a few (<10), some (10 to <12) and many (≥12). Psychological distress was ascertained from the five item Mental Health Index, a subscale of the SF-36[13] where respondents were asked how often (all, most, good bit, some, little, or none) they experienced five mental states in the last four weeks (have you been a very nervous person? Have you felt so down in the dumps that nothing could cheer you up? Have you felt calm and peaceful? Have you felt downhearted and blue? Have you been a happy person?). Psychological distress was classified intro three levels of distress: high (≤52), moderate (53 to 72) and low (≥73). BMI was grouped into four groups: underweight (<18.5), normal weight (18.5 to <25), overweight (25 to <30) and obesity (≥30).

Health services covariates included having visited a general practitioner or family doctor visit (yes/no), specialist visit (yes/no) and hospitalization (yes/no) in the last 12 months.

### Statistical analysis

Descriptive statistics of respondents' characteristics were reported using weighted proportions. These weights were used to account for complex survey design and non-participating bias. Differences between 2007 and 2012 were tested using unweighted chi-square test. Poisson regression models with robust variance estimators were used to estimate adjusted prevalence ratios (PR) and 95% confidence intervals (95%CI). Variance inflation factors were used to assess potential collinearity among socioeconomic variables and collinearity was not detected. For the main analyses, models were stratified by year and were adjusted for education, household income, employment status, demographic factors, health status, and health services use as described above. These variables were *a priori* considered given their potential associations with screening. [14] For socioeconomic indicators, different coding schemes were examined (education using three to five levels, income as a continuous versus nominal variable, employment in three versus two levels, occupational class in four versus six levels) to check robustness of results and results were similar (data not shown). Trends between 2007 and 2012 were tested by adding a wave (2012 *vs*. 2007) and predictor product term models. Trends were examined for the three CRC screening definitions described above. A model restricted to employed adults was conducted to examine the association between occupational class and CRC screening. All analyses were conducted with SPSS 22 and STATA 11.

### Sensitivity analyses

Since individuals aged 50–59 years did not have a full 10 years in which they could have received endoscopy, we conducted sensitivity analyses restricted to respondents’ aged 60–75 years. The same analyses were conducted with respondents’ aged 52–75 for hemoccult utilization. We also conducted additional analyses accounting for supplemental insurance (no, half-private or private) to see if the relationship between household income and CRC screening was altered.

## Results

5,946 and 7,224 respondents from the 2007 and 2012 SHIS were analyzed, respectively. Respondent socioeconomic characteristics varied between the two surveys. (**Table 1**) The median household income increased from $4,000 in 2007 to $4,130 in 2012. During the study period, there was a 5.2% decrease in respondents with secondary education and 7.2% increase in respondents reporting part or full-time employment. The proportion of divorced or separated respondents increased between 2007 and 2012. In terms of health indicators, obesity and psychological distress prevalence increased slightly between 2007 and 2012, while physical symptoms decreased during the period. Health services use also varied with time. The proportion of respondents reporting visiting a general practitioner within the past year declined from 81.5% in 2007 to 72.4% in 2012 whereas specialist visits increased slightly from 39.8% in 2007 to 43.0% in 2012 (**Table 1**).

**Table 1:** Respondent Characteristics by Survey Year Among Adults 50–75 years of age from Swiss Health Interview Survey 2007 and 2012 (n = 13,170).

	2007N = 5946	2012N = 7224	
Socioeconomic characteristics	N (%)^1^	N (%)^1^	p-value^2^
Household Income (USD)^3^			<0.001
≤2000	555(8.2)	514(7.2)	
2001–4000	2692(45.8)	3087(42.7)	
4001–6000	1708(28.9)	2264(31.7)	
>6000	991(17.2)	1359(18.5)	
Education			<0.001
Compulsory	801(11.4)	1029(14.0)	
Secondary	3631(60.9)	4129(55.7)	
Tertiary	1514(27.7)	2066(30.3)	
Employment status			<0.001
Out of the labour force	3077(46.1)	2964(38.9)	
Employed Full or Part-Time	2869(53.9)	4260(61.1)	
Occupational Class Among Employed (n = 7,129)			0.002
Liberal/Intermediate professions	1198(42.4)	1760(42.0)	
Non-manual professions	528(17.2)	676(15.2)	
Independent, artisan	516(17.3)	896(20.3)	
Overseer, qualified worker and skilled worker	627(23.1)	928(22.5)	
**Sociodemographic characteristics**			
Age (years)			0.002
50–64	3727(68.0)	4717(66.6)	
65–75	2219(32.0)	2507(33.4)	
Sex			<0.001
Male	2694(50.8)	3528(50.5)	
Female	3252(49.2)	3696(49.5)	
Marital status			<0.001
Single	571(6.7)	666(8.8)	
Married and Registered partnership	3552(72.0)	4960(67.7)	
Widow	806(8.2)	484(6.5)	
Divorced/Separated	1017(13.2)	1114(17.0)	
Citzenship			<0.001
Swiss	5532(88.9)	6434(86.4)	
Not Swiss	414(11.1)	790(13.6)	
Urban areas			<0.001
Metropolitan areas	2480(52.8)	3434(51.9)	
Medium size urban areas	1601(23.3)	1709(24.1)	
Small size urban areas	951(11.7)	1161(11.8)	
Rural areas	914(12.4)	920(12.2)	
**Health status**			
Self-rated health			<0.001
Very bad or bad	252(3.9)	360(4.6)	
So-so	819(12.6)	1265(17.3)	
Good	3861(66.3)	3370(47.3)	
Very good	1014(17.2)	2229(30.8)	
Body mass index			<0.001
Underweight	134(1.9)	163(2.0)	
Normal weight	2930(48.5)	3315(45.3)	
Overweight	2180(38.2)	2724(38.4)	
Obesity	702(11.3)	1022(14.2)	
Physical symptoms^4^			<0.001
No, a few	2255(41.4)	3363(49.1)	
Some	1990(35.3)	2172(30.9)	
Important	1427(23.3)	1429(20.0)	
Psychological distress^5^			0.010
High	240(3.5)	347(4.5)	
Moderate	675(10.8)	909(12.8)	
Low	4935(85.8)	5892(82.7)	
Currently smoking			0.523
Yes	1394(23.3)	1728(23.6)	
No	4552(76.7)	5496(76.4)	
Hospitalization last 12 months			0.577
No	5156(87.2)	6288(87.7)	
yes	790(12.8)	936(12.3)	
**Health service use**			
General practitioner visit(s) past 12 months			<0.001
No	1060(18.5)	1951(27.6)	
Yes	4886(81.5)	5273(72.4)	
Specialist visit(s) past 12 months			<0.001
No	3571(60.2)	4064(57.0)	
Yes	2375(39.8)	3160(43.0)	

^1^ Proportions are weighted.

^2^ Unweighted Pearson Chi-square test.

^3^ In October 2014, $1US Dollar = 1 CHF = 0.8 EUR.

^4.^Missing on 570 respondents

^5^ Missing on 208 respondents.

### Prevalence and trends of CRC screening reasons

**Fig 1:**
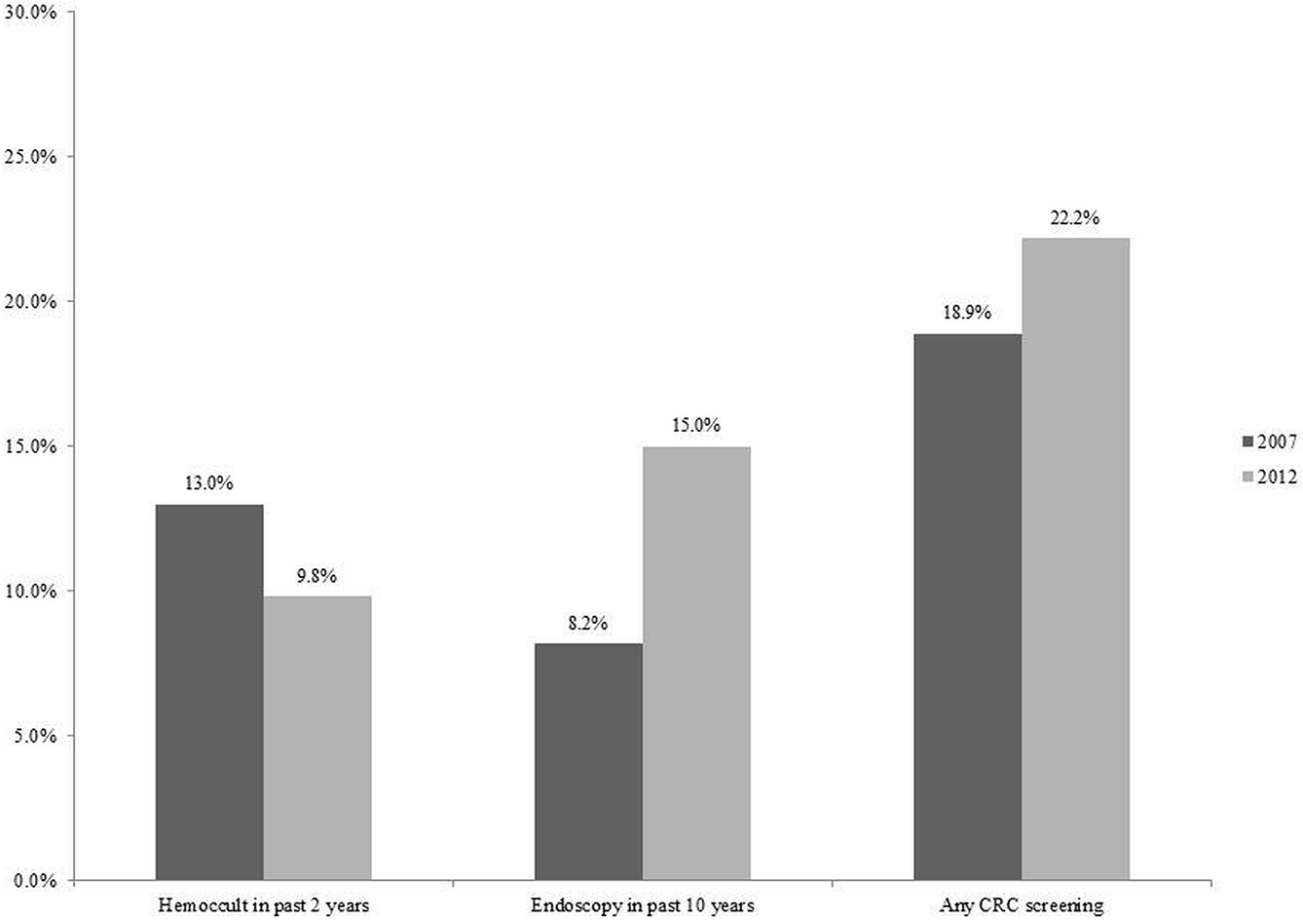
Colorectal Cancer Screening Weighted Prevalence among Respondents Aged 50–75 years of age from Swiss Health Interview Survey 2007–2012. Hemoccult in past 2 years 2007 vs 2012 (p-value = 0.002). Endoscopy in past 10 years 2007 vs 2012 (p-value<0.001). Any CRC screening (Hemoccult in past 2 years or endoscopy in past 10 years or both) 2007 vs 2012 (p-value = 0.036). P-values are adjusted for education, household income, employment, age, sex, marital status, citizenship, urban/rural status, health status and health care use.

Overall, CRC screening from either modality increased from 18.9% in 2007 to 22.2% in 2012 (adjusted p-value = 0.036). (**Fig 1**) This increase was due to growing endoscopy utilization, which rose from 8.2% in 2007 to 15.0% in 2012 (adjusted p-value <0.001). During the same period, hemoccult utilization decreased from 13.0% to 9.8% (adjusted p-value = 0.002). (**Fig 1**) The prevalence of CRC screening from either modality was 10% higher in 2012 compared to 2007 (PR = 1.10, 95%CI: 1.01–1.21) after adjusting for socioeconomic as well as demographic, health status and health utilization factors. The adjusted for prevalence of endoscopy was 44% higher in 2012 compared to 2007 (PR = 1.44, 95%CI: 1.26–1.65) whereas the adjusted prevalence of hemoccult use was 18% lower (PR = 0.82, 95%CI: 0.73–0.93).

### Socioeconomic determinants of CRC screening and temporal trends

#### Income

In 2007, the prevalence of CRC screening was 24.5% among respondents with higher income (>$6,000) and 10.5% among respondents with low income (<$2,000). In 2012, the corresponding percentages were 28.6% and 16.0%. (**S1 Table**). Household income remained positively associated with any CRC screening after adjusting for education, employment status, demographics, health status, and health service use. In adjusted analyses, CRC screening prevalence was 37% higher (not significantly) in high income respondents (>$6,000) compared to low income respondents (≤$2,000) in 2007 (PR = 1.37, 95%CI:0.96–1.96). In 2012, CRC screening was 45% higher in high *vs*. low income respondents (PR = 1.45, 95%CI:1.09–1.92) (**Table 2**). However, the test for temporal trend in CRC screening by household income was not significant (p-value for trend = 0.397). When endoscopy and hemoccult were examined separately, high income was associated with endoscopy use in 2007 and 2012 (2007 PR = 1.78, 95%CI: 1.01–3.12 and 2012 PR = 1.75, 95%CI: 1.21–2.54), but there was no association between income and hemoccult use in either year after adjusting for covariates.

**Table 2:** Adjusted and weighted prevalence ratios of colorectal cancer screening among adults aged 50–75 from the Swiss Health Interview Survey 2007 and 2012 (n = 13,170).

	Hemoccult test in past 2 years					Endoscopy in thepast 10 years					Any CRC Screening				
	2007		2012		p-value for trend^1^	2007		2012		p-value for trend^1^	2007		2012		p-value for trend^1^
	PR^2^	95%CI	PR^2^	95%CI		PR^2^	95%CI	PR^2^	95%CI		PR^2^	95%CI	PR^2^	95%CI	
**Socioeconomic characteristics**															
Household income (USD) (ref:≤2,000					0.880					0.221					0.397
2001–4000	1.11	0.73–1.70	0.97	0.68–1.39		1.29	0.80–2.08	1.11	0.79–1.56		1.10	0.81–1.51	1.07	0.83–1.38	
4001–6000	1.21	0.78–1.88	1.04	0.71–1.52		1.26	0.75–2.11	1.35	0.95–1.92		1.19	0.86–1.66	1.23	0.94–1.60	
>6001	1.23	0.76–1.97	1.05	0.69–1.60		1.78	1.01–3.12	1.75	1.21–2.54		1.37	0.96–1.96	1.45	1.09–1.92	
Education (ref: compulsory)					0.519					0.337					0.657
secondary	0.98	0.69–1.38	0.99	0.75–1.31		0.87	0.60–1.26	0.89	0.69–1.14		1.02	0.78–1.32	0.92	0.76–1.11	
tertiary	1.10	0.75–1.60	1.27	0.83–1.53		0.84	0.56–1.27	0.91	0.69–1.20		1.02	0.76–1.36	0.96	0.78–1.19	
Employed (ref:unemployed)	0.73	0.56–0.94	1.08	0.84–1.37	0.038	1.00	0.73–1.38	0.92	0.75–1.12	0.896	0.85	0.70–1.04	0.97	0.83–1.13	0.418
**Sociodemographiccharacteristics**															
Age 65–75 (ref: 50-64y)	0.93	0.71–1.21	1.15	0.89–1.47	0.856	1.32	0.97–1.80	1.31	1.07–1.60	0.726	1.06	0.87–1.29	1.21	1.04–1.41	0.876
Women	0.77	0.62–0.97	0.62	0.51–0.76	0.014	0.79	0.61–1.03	0.85	0.72–1.00	0.498	0.79	0.66–0.93	0.80	0.71–0.91	0.864
Married(ref: not married)	0.87	0.71–1.06	1.27	1.04–1.56	0.006	1.14	0.88–1.46	1.12	0.95–1.33	0.643	0.97	0.83–1.14	1.17	1.03–1.33	0.146
Not Swiss (ref: Swiss)	0.94	0.65–1.36	1.49	1.16–1.91	0.016	1.26	0.77–2.05	1.16	0.90–1.50	0.855	1.16	0.87–1.56	1.20	1.00–1.43	0.520
Urban areas (ref: Metro areas)					0.080					0.001					0.007
Medium size urban areas	1.00	0.80–1.24	0.92	0.75–1.12		0.75	0.56–1.01	0.87	0.74–1.03		0.92	0.78–1.10	0.90	0.79–1.02	
Small size urban areas	0.92	0.69–1.23	0.78	0.60–1.00		0.84	0.61–1.16	1.00	0.82–1.22		0.86	0.69–1.08	0.87	0.74–1.02	
Rural areas	1.01	0.74–1.38	0.65	0.45–0.93		1.61	1.21–2.15	0.93	0.71–1.21		1.24	1.00–1.52	0.84	0.69–1.05	
**Health services**															
GP visit in past 12m (ref: No)	2.27	1.55–3.22	2.26	1.73–2.96	0.952	2.40	1.54–3.75	1.42	1.16–1.74	0.094	2.23	1.66–2.98	1.71	1.45–2.02	0.260
Specialist visit in past 12 m (ref: No)	1.13	0.90–1.41	1.01	0.84–1.21	0.706	1.04	0.80–1.36	1.42	1.20–1.67	0.110	1.09	0.92–1.29	1.21	1.07–1.37	0.300

1. P-value for time-trend were estimated as follow: for each predictor (education, income, etc.), we estimated separately one multivariate model including all predictors plus the interaction term between the predictor and the wave. We reported only the p-value. 2. Prevalence ratios are adjusted for all variables in the table as well as for health statues which included self-rated health, body mass index, physical symptoms, psychological distress, hospitalization and smoking. Ref: reference category

#### Education, employment status and occupational class

Education and employment were not independently associated with CRC screening in adjusted analyses (**Table 2**). However, in 2007, hemoccult was lower among employed compared unemployed respondents, but no association was observed in 2012 (**Table 2**). In analyses restricted to respondents in the labor force (N = 7,129, 54.1% of the sample), independent (PR = 1.53, 95%CI: 1.09–2.16), non-manual employee (PR = 1.49, 95%CI:1.00–2.22), and superior professions (PR = 1.49, 95%CI:1.07–2.06) had significantly higher endoscopy utilization in 2012 compared to respondents with manual professions (**S2 Table**). This association was not apparent in 2007, however a temporal trend was not significant (p-value for trend = 0.104) (**S2 Table**).

### Other sociodemographic factors

Rural residence was associated with increased CRC screening (hemoccult or colonoscopy) compared to urban residence in 2007 (PR = 1.24, 95%CI 1.00–1.52), but not in 2012 (p-value for trend = 0.007) (**Table 2**). A similar pattern was observed when endoscopy and hemoccult were considered separately, however, the test for trend was only significant for endoscopy. Age and marital status were generally not associated with CRC screening, though married respondents had higher hemoccult prevalence in 2012 compared to 2007compared to their single/divorced/widowed counterparts (p-value for trend = 0.006)–a trend confirmed in analyses restricted to respondents in the labor force (**S2 Table**). Women were less likely to receive CRC screening compared to men and their use of hemoccult declined significantly over time (p-value for trend = 0.014).

### Health service use

Health services use, as measured by visiting a general practitioner in the past 12 months, was associated with higher prevalence of CRC screening overall in 2007 and 2012(**Table 2**). Visiting a general practitioner was also positively associated when hemoccult and endoscopy were analyzed separately. Visiting a specialist, including a gastroenterologist, was significantly related to endoscopy use in both 2007 and 2012.

### Sensitivity analyses

In sensitivity analyses restricted to adults aged 52–75 or 60–75, results were generally similar to those presented in our primary results among those aged 50–75, though among older (60–76) respondents, GP visits were not associated with endoscopy use (data not shown). However, adjustment for supplemental insurance (no, half-private or private) attenuated to the association between income and CRC screening (data not shown).

## Discussion

This study is the first to examine nationwide CRC screening trends in Switzerland. While CRC screening prevalence increased between 2007 and 2012, CRC screening utilization remains low, especially among adults with low income. The growth in CRC screening prevalence was due to greater use of endoscopy, which is in line with screening patterns in the United States. [15, 16] Yet, CRC screening prevalence in Switzerland (22%) is lower than the US where 58% of eligible adults are up to date with CRC screening. [17] Additionally, CRC screening prevalence in Switzerland, which does not have an organized CRC screening program, is considerably lower than in other European countries with organized screening programs [18–20] but similar to European countries (e.g: Belgium, Netherlands and Denmark) without organized screening programs. [20] Our CRC screening prevalence estimates were similar to other previous population-based estimates, but lower than surveys among hospital series, which is likely due to differences in study populations and we excluded diagnostic hemoccult and endoscopies. [10, 21]

Disparities in CRC screening by income persisted between 2007 and 2012 where adults with higher incomes had greater CRC screening utilization. These findings are consistent with other reports in Europe [18, 22], including a previous Swiss study.[4] The association between income and CRC screening in our study is likely due to a number of factors, including more financial barriers. Despite universal health care in Switzerland where healthcare costs, medical coverage and life expectancy are among the highest in the world, out of pocket expenses and health insurance premiums, which increased by 18% between 2007 and 2012, are substantial.[23] For example, during our study period, individuals were responsible for a 10% co-payment (up to annual limit of 700 CHF) for screening-related CRC tests after their annual deductible (which ranges from 300–2,500 CHF) was met, though patients/physicians may have been motivated to misrepresent symptoms in order to have their tests deemed diagnostic, and thus covered by insurance during this time. [4] A recent study reports that approximately 13% of Swiss forgo healthcare for economic reasons and this proportion is much higher among those in the lowest income (30%) compared to highest income bracket (4%).[5] Beginning July 2013, routine CRC screening (FOBT every 2 years and colonoscopy every 10 years) was fully in Switzerland covered under basic compulsory healthcare coverage, however, it is not known how these changes have affected CRC screening utilization.

Though cost is likely a barrier to CRC screening among low-income respondents, there are several other ways in which income may influence CRC screening. A favorable attitude toward screening is an important predictor of cancer screening, which is not only lower among lower-income individuals, but has been shown to mediate the association between income and cancer screening. [24] Furthermore, lower income is associated with lower adherence to free and organized CRC screening programs in other European counties where individuals were mailed an at-home FOBT kit. [18, 22] Such national programs would in theory, minimize such barriers as time off work and transportation issues. [18, 22]

Income disparities in CRC screening utilization persisted over our 5-year study period. A previous Swiss study reported a growing, but not statistically significant, proportion of healthcare renunciation for financial reasons between 2007 and 2010 among low-income residents. [5] Despite the global financial crisis in 2008, the overall economic impact on Switzerland has been minimal as unemployment, average number of hours worked per week, and income remained stable between 2007 and 2012.[25] Though the overall economic condition in Switzerland is positive, there may be subgroups of the population that may equally experience the positive economic condition which is reflected in the continued CRC screening disparity by income.

In terms of other socioeconomic measures, we did not observe an independent association between education and CRC screening use. In contrast, North American studies report positive associations between CRC and education independent of income.[14, 26] Previous studies in Switzerland have not observed an association with education, CRC mortality[27] and frailty[28], which could be due to narrower ranges of education attainment in Switzerland. Additionally, employment status (unemployed versus employed) was not associated with CRC screening, which is consistent with other findings. [29, 30] Among employed respondents, CRC screening prevalence was higher among professionals relative to manual laborers. The difference was mostly based on the 2012 survey and was largely unexplained, though it may be due to residual confounding by income level. Additional research on whether these differences will increase in the future, or if it’s due to random fluctuation is warranted.

We observed notable temporal changes by geographic residence. In 2007, rural respondents had higher CRC screening prevalence compared to urban respondents, while the opposite was observed in 2012. Changes in physician density may explain temporal patterns. Though medical density has increased markedly since the 1950’s, [31] in 2002, the Swiss government froze new accreditations for private practice physicians for a period of three years in order to reduce healthcare expenditures [32], renewed this decision for a supplementary period of three years (until 2008)[31] but only for specialists, until 2011. Initial reports noted a decline in general practitioners,[33] with an increase after the "freezing" period (2009–2011)[34] however, the continuation of these effects during the time of our study and the potential differential impact on rural versus urban regions is unknown. Studies examining regional variations in medical demography [35–37] are based on the Swiss cantons, not on the urban/rural variable, limiting the possibility of comparison with the existing literature. Additionally, we did not observe an association between health status and CRC screening.

### Limitations and Strengths

There are several limitations of our study worth noting. We did not have information on family history of CRC which strongly influences CRC screening adherence.[14] Though the prevalence of CRC family history in Switzerland has not been reported, prevalence of CRC family history in the US (which has similarly high incidence rates as Switzerland[38]) is low (<10%).[39, 40] Therefore, the degree to which family history of CRC confounds our results is likely to be minor. Selection bias may also be an issue in our study as SHIS response rates ranged from 53–66%. Non-responders have lower socioeconomic status [8] and may also have lower CRC screening utilization, leading to underestimation of CRC screening disparities by income. However, our use of weighted prevalence ratios mitigates the magnitude of this bias. Despite efforts to ask respondents in lay language about CRC test, there may have been misclassification of receipt of colonoscopy or hemoccult due to respondents not understanding the question or having inaccurate recall. Validation studies of self-reported cancer screening indicate that respondents may overestimate screening, though the degree of CRC screening misclassification is moderate. [41] Additionally, we were unable to differentiate between sigmoidoscopy and colonoscopy, which have different recommended scheduling (5 versus 10 years). However, it is reasonable to assume that the majority of endoscopies performed were colonoscopies as a previous Swiss study reported very low utilization of sigmoidoscopy.[10] Income data from the SHIS has not been validated and respondents may have overestimated their income. Furthermore, we excluded 1,697 (10.9%) respondents due to missing information on screening, sociodemographic, health status, and health service use, which may introduce selection bias though the proportion of respondents excluded due to missing information (10.9%) is small, limiting the magnitude of bias.

Despite these limitations our study has several strengths including our ability to differentiate between screening and diagnostic procedure and to assess various dimensions of socioeconomic position in a large population-based sample. Additionally, all analyses were weighted and corrected for sampling strategy which minimizes the risk of non-answer bias by sociodemographic factors and health status. This increases our confidence for population-based estimates of CRC screening in Switzerland. Additionally, we were able to adjust for many known risk factors for CRC cancer including smoking and obesity.

## Conclusion

This study is the first to examine nationwide changes CRC screening in Switzerland. While CRC screening prevalence in Switzerland increased from 2007 to 2012, CRC screening utilization remains insufficient. Additionally, low income Swiss residents had particularly inadequate CRC screening prevalence and this disparity persisted over time. These findings highlight the need for tailored interventions to increase the access to CRC screening, as well as increasing awareness of the benefits of CRC screening in the Swiss population, particularly among low-income residents. Additionally, the impact of adding CRC screening as a covered benefit to the basic Swiss health insurance plans in 2013 needs to be determined.

## Supporting Information

S1 Table Weighted prevalence of colorectal cancer screening among adults aged 50–75 from the Swiss Health Interview Survey (SHIS) 2007 and 2012 (n = 13,170). (DOCX)

S2 Table Adjusted and weighted prevalence ratios of colorectal cancer screening (for screening reasons) among adults aged 50–75 in the labor force from the Swiss Health Interview Survey (SHIS) 2007 and 2012 (n = 7,129). (DOCX)
