# Supplementary material for: Colorectal Cancer Screening in Switzerland: Cross-Sectional Trends (2007-2012) in Socioeconomic Disparities
Source: PLoS One. 2015 Jul 6;10(7):e0131205. doi: 10.1371/journal.pone.0131205 (PMC4492507; doi:10.1371/journal.pone.0131205)
Supplement: S1 Table — (DOCX) [file pone.0131205.s001.docx]

**Table S1. Weighted prevalence of colorectal cancer screening among adults aged 50-75 from the Swiss Health Interview Survey (SHIS) 2007 and 2012 (n=13,170)**

|  |  | **Hemoccult test in the past 2 years** | | |  | **Endoscopy in the past 10 years** | | |  | **Any CRC Screening** | | |
| --- | --- | --- | --- | --- | --- | --- | --- | --- | --- | --- | --- | --- |
|  |  | 2007  N=6224 | 2012  N=7671 |  |  | 2007  N=6224 | 2012  N=7671 |  |  | 2007  N=6224 | 2012  N=7671 |  |
|  |  | %^1^ | %^1^ | p-value^2^ |  | %^1^ | %^1^ | p-value^2^ |  | %^1^ | %^1^ | p-value^2^ |
| **Socioeconomic characteristics** |  |  |  |  |  |  |  |  |  |  |  |  |
| Household income in CHF^3^ |  |  |  | 0.001 |  |  |  | <0.001 |  |  |  | <0.001 |
| ≤2000 |  | 6.3 | 8.1 |  |  | 4.8 | 10.1 |  |  | 10.5 | 16.0 |  |
| 2001-4000 |  | 12.3 | 9.5 |  |  | 6.6 | 12.7 |  |  | 17.1 | 20.1 |  |
| 4001-6000 |  | 14.4 | 10.0 |  |  | 8.6 | 15.3 |  |  | 20.9 | 22.6 |  |
| >6000 |  | 15.8 | 11.0 |  |  | 13.4 | 21.8 |  |  | 24.5 | 28.6 |  |
| Education |  |  |  | <0.001 |  |  |  | <0.001 |  |  |  | <0.001 |
| compulsory |  | 8.9 | 9.5 |  |  | 5.4 | 13.4 |  |  | 12.8 | 20.2 |  |
| secondary |  | 12.5 | 9.0 |  |  | 7.2 | 14.3 |  |  | 17.7 | 20.9 |  |
| tertiary |  | 15.9 | 11.5 |  |  | 11.6 | 17.1 |  |  | 24.1 | 25.4 |  |
| Employment status |  |  |  | 0.002 |  |  |  | <0.001 |  |  |  | <0.001 |
| Unemployed |  | 14.4 | 10.7 |  |  | 9.0 | 18.0 |  |  | 20.8 | 25.8 |  |
| Employed |  | 11.9 | 9.3 |  |  | 7.6 | 13.2 |  |  | 17.4 | 19.9 |  |
| Occupational class of employed |  |  |  | 0.001 |  |  |  | <0.001 |  |  |  | <0.001 |
| Superior and intermediate professions |  | 14.6 | 10.3 |  |  | 9.6 | 16.2 |  |  | 21.3 | 23.8 |  |
| Employee, non-manual professions |  | 8.9 | 7.4 |  |  | 6.9 | 8.4 |  |  | 14.2 | 14.6 |  |
| Independent, artisan |  | 8.0 | 8.1 |  |  | 6.3 | 12.6 |  |  | 13.3 | 17.8 |  |
| Overseer, qualified worker, skilled worker |  | 12.0 | 9.6 |  |  | 5.2 | 11.2 |  |  | 15.6 | 18.1 |  |
| **Sociodemographic characteristics** |  |  |  |  |  |  |  |  |  |  |  |  |
| Age |  |  |  | <0.001 |  |  |  | <0.001 |  |  |  | <0.001 |
| 50-64 years |  | 12.0 | 8.7 |  |  | 7.2 | 12.9 |  |  | 17.2 | 19.5 |  |
| 65-75 years |  | 15.2 | 12.0 |  |  | 10.4 | 19.2 |  |  | 22.6 | 27.5 |  |
| Sex |  |  |  | <0.001 |  |  |  | <0.001 |  |  |  | <0.001 |
| Male |  | 16.0 | 12.2 |  |  | 9.8 | 17.7 |  |  | 22.4 | 26.1 |  |
| Female |  | 10.0 | 7.4 |  |  | 6.6 | 12.3 |  |  | 15.1 | 18.1 |  |
| Marital status |  |  |  | 0.053 |  |  |  | 0.001 |  |  |  | <0.001 |
| Single |  | 14.0 | 6.7 |  |  | 8.6 | 11.8 |  |  | 19.6 | 16.9 |  |
| Married |  | 13.5 | 10.5 |  |  | 8.5 | 15.6 |  |  | 19.7 | 23.3 |  |
| Widow |  | 9.6 | 11.3 |  |  | 7.9 | 18.2 |  |  | 16.7 | 25.2 |  |
| Divorced/separated |  | 12.0 | 8.1 |  |  | 6.6 | 13.3 |  |  | 15.9 | 19.1 |  |
| Citizenship |  |  |  | 0.231 |  |  |  | 0.705 |  |  |  | 0.767 |
| Swiss |  | 13.0 | 9.9 |  |  | 8.2 | 15.6 |  |  | 18.9 | 22.9 |  |
| Not Swiss |  | 13.0 | 9.2 |  |  | 7.9 | 11.7 |  |  | 18.9 | 17.9 |  |
| Urban areas |  |  |  | <0.001 |  |  |  | <0.001 |  |  |  | <0.001 |
| Metropolitan areas |  | 14.3 | 10.3 |  |  | 9.7 | 16.0 |  |  | 21.3 | 23.4 |  |
| Medium size urban areas |  | 13.9 | 11.0 |  |  | 6.2 | 14.6 |  |  | 18.3 | 22.8 |  |
| Small size urban areas |  | 8.5 | 9.1 |  |  | 6.2 | 14.5 |  |  | 13.7 | 20.9 |  |
| Rural areas |  | 10.2 | 6.2 |  |  | 7.4 | 12.3 |  |  | 15.2 | 17.2 |  |
| **Health status** |  |  |  |  |  |  |  |  |  |  |  |  |
| Self-rated health |  |  |  | 0.093 |  |  |  | 0.221 |  |  |  | 0.033 |
| Very bad or bad |  | 4.1 | 10.4 |  |  | 6.2 | 10.0 |  |  | 9.4 | 17.9 |  |
| So-so |  | 9.5 | 9.3 |  |  | 7.1 | 13.9 |  |  | 15.0 | 21.0 |  |
| Good |  | 13.9 | 9.9 |  |  | 7.9 | 15.8 |  |  | 19.7 | 22.6 |  |
| Very Good |  | 14.5 | 9.8 |  |  | 10.6 | 15.3 |  |  | 21.0 | 22.9 |  |
| Body mass index |  |  |  | 0.001 |  |  |  | 0.005 |  |  |  | <0.001 |
| Underweight |  | 2.5 | 7.1 |  |  | 5.2 | 14.3 |  |  | 7.1 | 18.2 |  |
| Normal weight |  | 12.8 | 8.6 |  |  | 7.9 | 14.2 |  |  | 18.7 | 20.6 |  |
| Overweight |  | 14.5 | 10.9 |  |  | 9.2 | 16.2 |  |  | 20.8 | 24.2 |  |
| Obesity |  | 11.1 | 10.9 |  |  | 6.8 | 14.6 |  |  | 15.7 | 22.3 |  |
| Physical symptoms (missings 570) |  |  |  | 0.125 |  |  |  | 0.001 |  |  |  | 0.002 |
| No, a few |  | 13.8 | 10.1 |  |  | 8.4 | 16.1 |  |  | 19.5 | 23.5 |  |
| Some |  | 13.1 | 10.3 |  |  | 8.1 | 15.6 |  |  | 18.9 | 22.7 |  |
| Important |  | 11.6 | 8.2 |  |  | 8.8 | 11.9 |  |  | 18.4 | 18.0 |  |
| Psychological distress (missings 208) |  |  |  | 0.040 |  |  |  | 0.018 |  |  |  | <0.001 |
| High |  | 6.2 | 10.9 |  |  | 4.0 | 10.3 |  |  | 9.2 | 17.0 |  |
| Moderate |  | 11.2 | 7.9 |  |  | 8.4 | 15.0 |  |  | 17.1 | 20.7 |  |
| low |  | 13.6 | 10.1 |  |  | 8.4 | 15.3 |  |  | 19.7 | 22.7 |  |
| Currently smoking |  |  |  | 0.761 |  |  |  | 0.001 |  |  |  | 0.021 |
| Yes |  | 13.1 | 9.9 |  |  | 9.2 | 11.7 |  |  | 19.1 | 19.3 |  |
| No |  | 13.0 | 9.8 |  |  | 7.9 | 16.1 |  |  | 18.9 | 23.1 |  |
| Hospitalization last 12 months |  |  |  | 0.591 |  |  |  | <0.001 |  |  |  | 0.001 |
| No |  | 13.2 | 9.8 |  |  | 7.9 | 14.9 |  |  | 18.8 | 22.0 |  |
| Yes |  | 12.0 | 9.7 |  |  | 10.1 | 15.8 |  |  | 19.7 | 23.3 |  |
| **Health services characteristics** |  |  |  |  |  |  |  |  |  |  |  |  |
| General practitioner or family doctor visit(s) in the last 12 months |  |  |  | <0.001 |  |  |  | <0.001 |  |  |  | <0.001 |
| No |  | 8.8 | 5.7 |  |  | 3.9 | 10.8 |  |  | 11.6 | 15.3 |  |
| Yes |  | 14.0 | 11.4 |  |  | 9.2 | 16.6 |  |  | 20.6 | 24.8 |  |
| Specialist visit(s) in the last 12 months |  |  |  | <0.001 |  |  |  | <0.001 |  |  |  | <0.001 |
| No |  | 12.4 | 8.4 |  |  | 6.6 | 11.8 |  |  | 17.2 | 18.5 |  |
| Yes |  | 14.0 | 11.7 |  |  | 10.7 | 19.2 |  |  | 21.6 | 27.1 |  |

^1^ Proportions are weighted.

^2^ Unweighted Pearson Chi-square test

^3^ In October 2014, $1US Dollar=1 CHF= 0.8 EUR
