# Supplementary material for: Colorectal Cancer Screening in Switzerland: Cross-Sectional Trends (2007-2012) in Socioeconomic Disparities
Source: PLoS One. 2015 Jul 6;10(7):e0131205. doi: 10.1371/journal.pone.0131205 (PMC4492507; doi:10.1371/journal.pone.0131205)
Supplement: S2 Table — (DOCX) [file pone.0131205.s002.docx]

**Table S2 Adjusted and weighted prevalence ratios of colorectal cancer screening (for screening reasons) among adults aged 50-75 in the labor force from the Swiss Health Interview Survey (SHIS) 2007 and 2012 (n=7,129)**

|  | Hemoccult test in the past 2 years | | | | | Endoscopy in the past 10 years | | | | | Any CRC Screening | | | | |
| --- | --- | --- | --- | --- | --- | --- | --- | --- | --- | --- | --- | --- | --- | --- | --- |
|  | 2007 | | 2012 | | p-value for trend^a^ | 2007 | | 2012 | | p-value for trend^a^ | 2007 | | 2012 | | p-value for trend^a^ |
|  | PR^b^ | 95%CI | PR^b^ | 95%CI |  | PR^b^ | 95%CI | PR^b^ | 95%CI |  | PR^b^ | 95%CI | PR^b^ | 95%CI |  |
| **Socioeconomic characteristics** |  |  |  |  |  |  |  |  |  |  |  |  |  |  |  |
| Household income (ref: ≤2000) |  |  |  |  | 0.480 |  |  |  |  | 0.667 |  |  |  |  | 0.907 |
| 2001-4000 | 1.51 | 0.55-4.16 | 1.69 | 0.79-3.59 |  | 1.00 | 0.38-2.65 | 1.21 | 0.65-2.27 |  | 1.09 | 0.55-2.16 | 1.27 | 0.79-2.07 |  |
| 4001-6000 | 1.53 | 0.55-4.25 | 1.94 | 0.90-4.17 |  | 0.85 | 0.32-2.24 | 1.66 | 0.91-3.04 |  | 1.07 | 0.54-2.14 | 1.57 | 0.98-2.54 |  |
| >6000 | 1.43 | 0.49-4.19 | 1.57 | 0.70-3.49 |  | 1.45 | 0.53-3.97 | 1.78 | 0.96-3.33 |  | 1.25 | 0.61-2.58 | 1.53 | 0.94-2.51 |  |
| Education  (ref: compulsory) |  |  |  |  | 0.580 |  |  |  |  | 0.589 |  |  |  |  | 0.200 |
| secondary | 1.24 | 0.66-2.34 | 0.95 | 0.61-1.50 |  | 3.89 | 1.65-9.13 | 0.81 | 0.55-1.19 |  | 1.78 | 1.06-2.99 | 0.82 | 0.61-1.09 |  |
| tertiary | 1.43 | 0.75-2.73 | 1.20 | 0.74-1.93 |  | 3.63 | 1.48-8.93 | 0.86 | 0.56-1.32 |  | 1.92 | 1.11-3.31 | 0.89 | 0.65-1.22 |  |
| Occupational class (ref. overseer, qualified worker, skilled worker) |  |  |  |  | 0.483 |  |  |  |  | 0.104 |  |  |  |  | 0.295 |
| Independent, artisan | 0.93 | 0.54-1.61 | 1.13 | 0.78-1.63 |  | 0.88 | 0.47-1.66 | 1.53 | 1.09-2.16 |  | 0.98 | 0.65-1.46 | 1.35 | 1.05-1.74 |  |
| Employee, non-manual professions | 0.78 | 0.45-1.35 | 1.40 | 0.89-2.18 |  | 0.91 | 0.50-1.65 | 1.49 | 1.00-2.22 |  | 0.87 | 0.58-1.30 | 1.53 | 1.15-2.05 |  |
| Superior and intermediate professions | 1.21 | 0.79-1.86 | 1.20 | 0.82-1.77 |  | 0.84 | 0.51-1.38 | 1.49 | 1.07-2.06 |  | 1.03 | 0.74-1.43 | 1.45 | 1.14-1.85 |  |
| **Sociodemographic characteristics** |  |  |  |  |  |  |  |  |  |  |  |  |  |  |  |
| Age 65-75  (ref: 50-64y) | 1.09 | 0.62-1.92 | 1.55 | 1.16-2.08 | 0.273 | 1.77 | 1.07-2.94 | 1.50 | 1.14-1.97 | 0.585 | 1.29 | 0.89-1.85 | 1.45 | 1.19-1.76 | 0.715 |
| Women | 0.64 | 0.45-0.92 | 0.60 | 0.45-0.81 | 0.357 | 0.74 | 0.50-1.10 | 0.76 | 0.59-0.98 | 0.594 | 0.69 | 0.54-0.90 | 0.74 | 0.62-0.89 | 0.620 |
| Married  (ref: not married) | 0.80 | 0.58-1.09 | 1.35 | 1.02-1.78 | 0.008 | 0.90 | 0.61-1.33 | 1.05 | 0.83-1.32 | 0.710 | 0.82 | 0.64-1.04 | 1.17 | 0.98-1.39 | 0.041 |
| Not Swiss | 1.03 | 0.61-1.72 | 1.51 | 1.09-2.09 | 0.204 | 1.34 | 0.63-2.84 | 1.16 | 0.84-1.61 | 0.902 | 1.22 | 0.81-1.83 | 1.23 | 0.99-1.53 | 0.720 |
| Urban areas  (ref: Metro areas) |  |  |  |  | 0.790 |  |  |  |  | 0.082 |  |  |  |  | 0.203 |
| Medium size urban areas | 1.10 | 0.80-1.52 | 0.82 | 0.63-1.06 |  | 1.14 | 0.74-1.76 | 0.87 | 0.69-1.10 |  | 1.14 | 0.88-1.47 | 0.85 | 0.72-1.02 |  |
| Small size urban areas | 0.94 | 0.61-1.44 | 0.77 | 0.55-1.08 |  | 0.78 | 0.47-1.28 | 1.18 | 0.92-1.53 |  | 0.85 | 0.61-1.19 | 0.95 | 0.78-1.17 |  |
| Rural areas | 0.75 | 0.44-1.29 | 0.69 | 0.43-1.12 |  | 1.55 | 0.98-2.46 | 0.67 | 0.44-1.01 |  | 1.09 | 0.78-1.53 | 0.70 | 0.51-0.96 |  |
| **Health services uses characteristics** |  |  |  |  |  |  |  |  |  |  |  |  |  |  |  |
| GP visits past 12m (ref: No) | 2.70 | 1.74-4.19 | 2.45 | 1.77-3.40 | 0.939 | 2.01 | 1.13-3.58 | 1.48 | 1.14-1.91 | 0.789 | 2.22 | 1.55-3.19 | 1.86 | 1.51-2.29 | 0.959 |
| Specialist visits past 12 m (ref: No) | 1.12 | 0.79-1.59 | 1.02 | 0.80-1.29 | 0.921 | 0.89 | 0.59-1.34 | 1.54 | 1.22-1.93 | 0.052 | 0.97 | 0.74-1.26 | 1.24 | 1.04-1.46 | 0.100 |

^a.^ P-values for time-trend were estimated as follow: for each predictor (education, income, etc.), we estimated separately one multivariate model including all predictors plus the interaction term between the predictor and the wave. We reported only the p-value.

^b.^ Prevalence ratios are adjusted for all variables in the table as well as for health statuses which included self-rated health, body mass index, physical symptoms, psychological distress, hospitalization and smoking.
